# Transcribe this way: Rap1 confers promoter directionality by repressing divergent transcription

**DOI:** 10.1080/21541264.2019.1608716

**Published:** 2019-05-05

**Authors:** Andrew C.K. Wu, Folkert J. Van Werven

**Affiliations:** Cell Fate and Gene Regulation Laboratory, The Francis Crick Institute, London, UK

**Keywords:** Rap1, directionality, divergent, noncoding RNA, promoter, repression, yeast, steric hindrance, transcription factor

## Abstract

In eukaryotes, divergent transcription is a major source of noncoding RNAs. Recent studies have uncovered that in yeast, the transcription factor Rap1 restricts transcription in the divergent direction and thereby controls promoter directionality. Here, we summarize these findings, propose regulatory principles, and discuss the implications for eukaryotic gene regulation.

## Introduction

**Figure 1.**
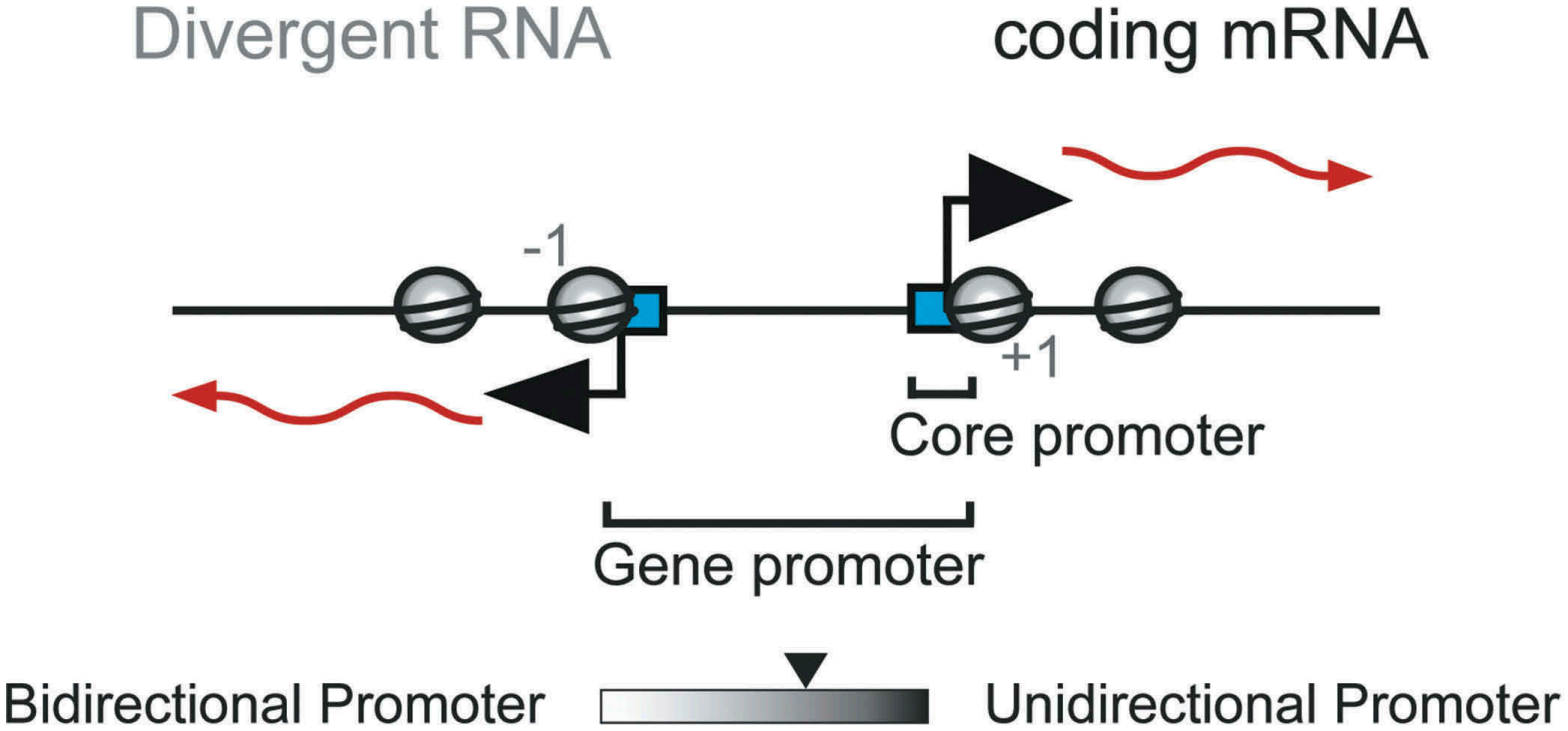
Schematic diagram of a bidirectional gene promoter. A bidirectional gene promoter comprises separate core promoters (blue boxes) for the coding messenger RNA (mRNA) and divergent RNA, arranged in opposite orientations. These core promoters are typically located at the edges of a nucleosome-depleted region, flanked by the +1 and −1 nucleosomes (gray circles). The relative amount of transcription from each core promoter dictates the overall “directionality” of the gene promoter. The scale bar illustrates that the output of eukaryotic gene promoters ranges widely: some promoters are more unidirectional, whereas others display more bidirectional transcription.

Eukaryotic gene promoters are inherently bidirectional [1]. This process, known as divergent or bidirectional transcription, generates upstream transcripts in the opposing direction to the coding gene from a distinct core promoter (Figure 1) [2]. The divergent transcripts produced are typically unstable and are a major source of noncoding RNAs [3,4].

The functions of divergent noncoding transcription, and the RNAs generated, are not well understood. Many divergent noncoding transcripts, but not all, are likely the non-functional products of “noisy” transcription [5,6]. Some roles for divergent transcription have been proposed. For example, divergent transcription may facilitate new gene formation. Non-functional enhancer RNAs and divergent transcripts can be co-opted for biological functions by evolutionary pressures [7]. Transcription at gene promoters is inherently bidirectional, but evolutionary forces shift bidirectional output towards unidirectional coding gene transcription over time [1]. Divergent transcripts themselves can also regulate gene expression directly in *cis* or in *trans* [8–11].

Mis-expression of divergent transcripts may compromise cellular fitness. In organisms like *Saccharomyces cerevisiae*, the distance between genes is relatively short. As a consequence, divergent transcription can overlap with neighboring genes and cause transcriptional interference [12]. If neighboring genes are oriented in tandem, for example, divergent transcripts can overlap with upstream genes as antisense long noncoding RNAs. Inappropriate noncoding transcription can also generate R-loops *in vivo*, which compromises genomic stability [13,14]. In addition, aberrant divergent transcription is wasteful and energetically costly for cells [15]. To complete the transcription cycle, many macromolecular machines are produced, assembled, and recruited to DNA. Therefore, cells must have robust mechanisms to limit the inappropriate expression of divergent transcripts.

Expression of divergent RNAs is controlled at multiple steps during gene expression. Pathways involved in chromatin structure, RNA termination, and RNA degradation play significant roles in limiting the accumulation of divergent transcripts [6]. For example, histone modifications like H3K56 (histone H3 lysine 56) acetylation and variants like H2A.Z regulate divergent transcription by modulating nucleosome assembly and remodeling [16,17]. In addition, control of TATA-binding protein activity can also limit divergent and pervasive transcription, as can RNA polymerase speed [18–20]. Divergent transcription of noncoding RNAs is also controlled by gene looping and chromatin conformation [21]. Finally, transcription termination and RNA turnover limit accumulation of aberrant transcripts. The Nrd1-Nab3-Sen1 (NNS) and premature polyadenylation signal (PAS) pathways in yeast and mammalian cells, respectively, terminate and degrade divergent promoter transcripts [22,23]. The Integrator complex is also likely involved in termination of divergent transcription [14]. Exosome and nonsense-mediated decay pathways degrade cryptic and divergent RNAs, limiting their expression [20,24,25]. Together, these pathways limit the presence of pervasive divergent RNAs.

## Rap1, a transcriptional activator that represses divergent transcription

We recently identified that the transcription factor Rap1 confers promoter directionality by specifically repressing initiation of divergent noncoding transcription [26]. Rap1 has multiple functions in yeast. This pioneer transcription factor is essential for telomere and hidden mating type silencing, and activates highly expressed ribosomal protein (RP) and glycolytic genes [27–29]. In parallel with a study by Challal *et al*., we showed that rapid depletion of Rap1 leads to widespread induction of divergent transcripts very close to sites where Rap1 is bound [26,30]. We validated this proximity-dependent effect of Rap1 binding through mutagenesis at representative RP gene and divergent promoters. Without Rap1, divergent transcripts can disrupt regulatory circuits controlling cell fate decisions or interfere with gene expression at mRNA and protein levels. These examples illustrate the importance of controlling promoter directionality at very active promoters. Thus, Rap1 represses divergent transcription at hundreds of highly expressed genes throughout the yeast genome.

How does Rap1 repress divergent transcription? We investigated whether chromatin regulators or co-repressors are required for Rap1-mediated transcriptional repression. We found that Rap1 and other chromatin regulators repress discrete divergent or cryptic antisense promoters at distinct genomic locations, and are not redundant. Rap1 most likely represses divergent transcription directly, because its cofactors and interacting partners do not inhibit divergent transcription from Rap1-regulated promoters.

In contrast, Rap1 does not repress divergent transcription from gene promoters as a transcriptional “roadblock”. Rap1 and a related transcription factor, Reb1, can terminate elongating RNA polymerase and prevent read-through transcription from interfering with downstream gene expression [31–33]. However, Rap1 binding sites at gene promoters are extremely close to divergent transcription start sites (TSSs) – within 0–50 base pairs (bp) – which would interfere with transcription initiation instead. In addition, there is no potential roadblock posed by Rap1 for most divergent transcripts, as most of their TSSs are upstream (not downstream) of promoter Rap1 binding sites. We tested this model experimentally by repositioning the Rap1 binding sites in the *RPL43B* promoter 400 bp downstream of the divergent transcript *IRT2* TSS in a potential “roadblock” position, and found that the divergent transcript was not effectively repressed. In addition, proximal Rap1 binding sites located upstream of an independent divergent TSS were already sufficient to repress divergent transcription [26]. Thus, Rap1 limits divergent transcript expression by regulating transcription initiation, not elongation. We propose that a stable physical association between Rap1 and its target sequences at promoters can achieve repression of divergent transcription. Therefore, Rap1 may specifically block or reduce the association of transcriptional activators and general transcription machinery to the divergent core promoter.

**Figure 2.**
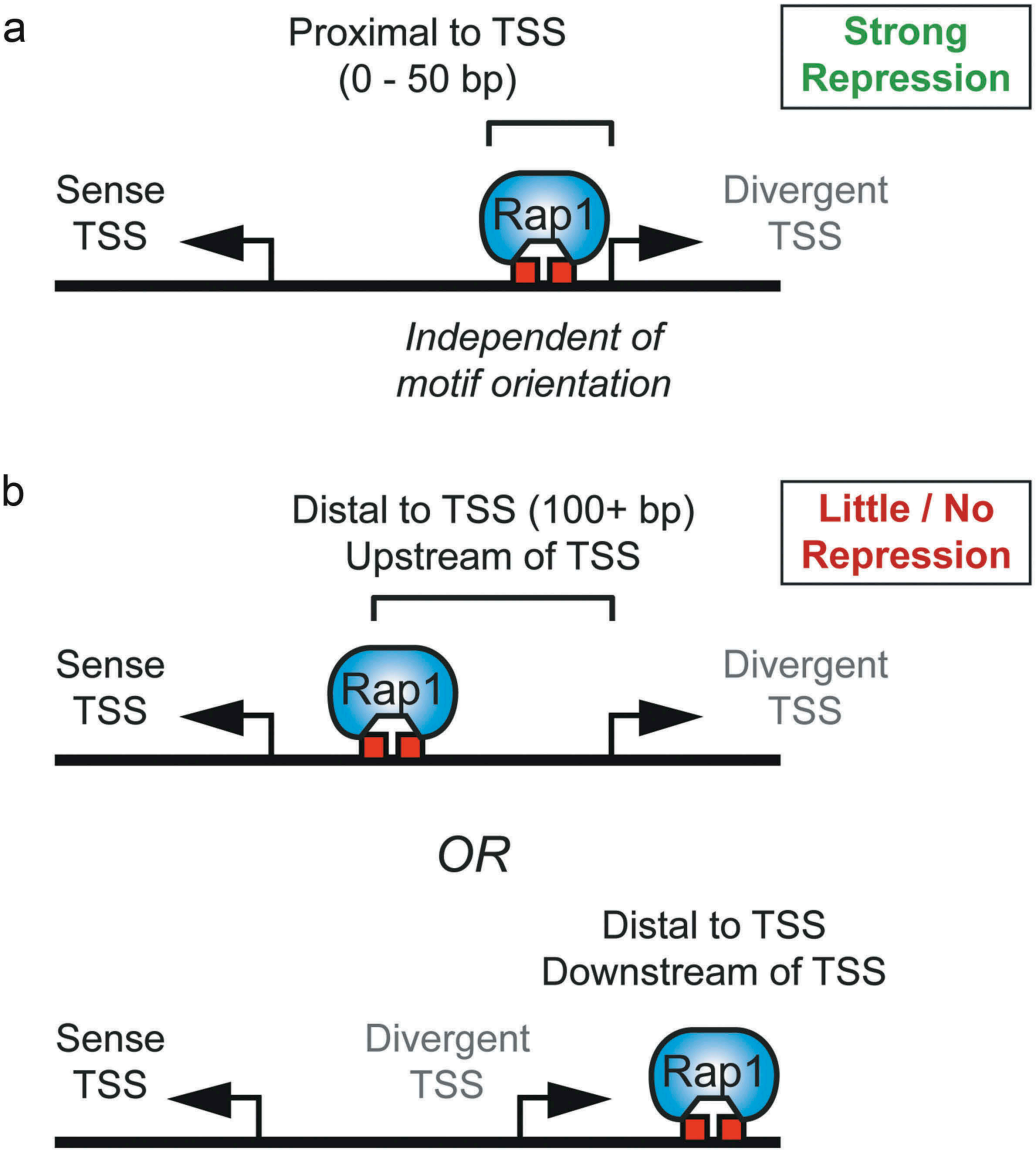
Requirements for steric hindrance of divergent transcription initiation. (a) Transcription factor binding site (red boxes) must be within ~50 bp of divergent transcription start site (TSS) for effective repression of transcription. Repression at proximal binding sites does not depend on specific Rap1 motif orientation. (b) Rap1 binding at distal sites, upstream or downstream of the divergent TSS, does not effectively limit the expression of divergent transcripts.

The steric hindrance model is attractive for several reasons. First, Rap1 is ideally positioned to restrict initiation of divergent transcription, typically within 50 bp of its binding sites. Rap1 binds at the 5ʹ (upstream) edge of the promoter nucleosome-depleted region (NDR), where divergent transcription initiates. Steric hindrance of the divergent core promoter is spatially limited and does not interfere with gene transcription in the coding direction. Rap1 binding sites *in vivo* are several hundred base pairs upstream of coding direction TSSs. If the Rap1 binding site(s) were repositioned in close proximity to the coding TSS, away from the divergent TSS, we would expect divergent transcription to increase while coding direction transcription would decrease. Second, the physical association of Rap1 with its target motif confers effective transcriptional repression independent of Rap1 motif orientation (Figure 2). Finally, Rap1 maintains a stable association with DNA during different cellular states, in contrast to other RP gene coactivators that dissociate from the promoter after stress [34]. To maintain repression of divergent noncoding transcription, Rap1 must stably bind its target motifs at promoters. Limiting the recruitment of basal transcription machinery could be an efficient way to limit divergent transcription at highly expressed genes.

The intrinsic features of Rap1-regulated genes may justify the use of this specialized mechanism to regulate divergent transcripts. Highly transcribed genes tend to have wider NDRs, and promoters activated by Rap1 are among the most active in yeast. Therefore, it is not surprising that Rap1-regulated promoters contain a wide NDR approximately 200–400 bp in length, compared to 150 bp for the average yeast promoter [35,36]. The wide NDR generated by Rap1, together with coactivators and chromatin remodelers, facilitates proper coding gene activation [27,28,37]. Without stringent control by Rap1 and its cofactors, open chromatin could allow inappropriate recruitment of RNA polymerase II. Subsequently, aberrant transcription could proceed in both directions from the distinct coding and divergent core promoters that occupy the outer borders of NDRs [38]. Rap1 depletion also shifts TSS usage upstream in the sense direction, which compromises coding gene expression in many cases [26,30]. We propose that Rap1 reduces the association of initiation factors, basal transcription machinery, and ATP-dependent chromatin remodellers at sequences surrounding the Rap1 binding site, to stimulate productive transcription in the protein-coding direction only. In other words, Rap1 is positioned to repress initiation of divergent transcription, while concurrently facilitating orderly recruitment of cofactors to drive transcription in the coding gene direction.

## Transcriptional repression using steric hindrance

The ability to repress transcription using steric hindrance is not unique to Rap1. Gene regulation through steric hindrance is widespread through all three domains of life, viruses, and can be recapitulated with synthetic repressors (Table 1). In bacteria, classic repressors such as LexA and Lac repressor inhibit transcription through the steric exclusion of RNA polymerase from gene promoters [39–41]. Direct repression usually targets the coding direction core promoter. Synthetic transcriptional repression systems like CRISPRi (CRISPR interference [CRISPR, clustered regularly interspaced short palindromic repeats]) or TALE repressors (TALE, transcription activator-like effector) also reconstitute direct steric repression of transcription initiation [42–45].

**Table 1.** Examples of transcriptional repression by steric hindrance in different organisms.

Factor	Species or Origin	Reference
**Bacteria**		
Trp repressor	*E. coli*	Kumamoto et al., 1987^[59]^
LexA repressor	*E. coli*	Little et al., 1981^[39]^
		Brent and Ptashne, 1981^[40]^
Lac repressor	*E. coli*	Sellitti et al., 1987^[41]^
**Archaea**		
MDR1 repressor	*A. fulgidus*	Bell et al., 1999^[60]^
LrpA repressor	*P. furiosus*	Brinkman et al., 2000^[61]^
Phr heat shock response regulator	*P. furiosus*	Vierke et al., 2003^[62]^
Eukaryotes		
AP2	*H. sapiens*	Getman et al., 1995^[63]^
Glucocorticoid receptor (GR)	*B. taurus*	Sakai et al., 1988^[64]^
Rap1, likely Reb1 & Abf1	*S. cerevisiae*	Wu et al., 2018^[26]^
		Challal et al., 2018^[30]^
**Viruses**		
cI and Cro	Lambda (λ) bacteriophage	Meyer et al., 1975^[65]^
		Johnson et al., 1978^[66]^
T antigen	SV40	Myers et al., 1981^[67]^
LBP-1 (host factor)	HIV-1	Kato et al., 1991^[68]^
**Synthetic systems**		
dCas9 (catalytic inactivated Cas9 mutant)	From *S. pyogenes*	Qi et al., 2013^[43]^
		Gilbert et al., 2013^[42]^
TALEs	From *Xanthamonas sp.*	Li et al., 2015^[45]^
		Clauß et al., 2017 ^[44]^

Some examples of transcriptional repression through steric hindrance are listed, from different sources including all three domains of life, viruses, and synthetic repression systems (not a comprehensive list).

We can only speculate about other transcription factors that repress divergent transcription *in vivo*. In *S. cerevisiae*, Rap1 has been co-opted to drive most RP gene expression by transcription factor motif substitution [46]. Other transcription factors such as Abf1, Reb1, Tbf1, and Cbf1 also share structural homology with the Rap1 DNA binding domain, possess “pioneer” nucleosome displacement activity, and drive RP gene expression in other fungal species [47,48]. These transcription factors may also fulfill the requirements for steric hindrance of divergent transcription; this hypothesis requires experimental validation. In higher eukaryotes, certain sequence-specific transcription factors may perform analogous roles in the regulation of divergent transcription. Recent work has assessed the contribution of chromatin states and core promoter sequence towards promoter directionality in metazoans [49]. A number of pioneer transcription factors that open DNA asymmetrically were also identified, belonging to the Klf/Sp, NFYA, Creb/ATF, and Zfp161 families [50]. These transcription factors are present at the edges of NDRs at promoters and enhancers, where divergent core promoters are also located, and thus are ideally positioned to repress divergent transcription.

Comparing closely or distantly related species can highlight key regulatory principles controlling the expression of divergent transcripts [1]. Some eukaryotes, like *Drosophila melanogaster*, were thought to have little to no divergent transcription [51]. However, technical advances in the detection of nascent transcription uncovered widespread expression of divergent transcripts, which are unstable in many cases [52,53]. In *Arabidopsis* seedlings, GRO-seq (global run-on sequencing) and NET-seq (native elongating transcript sequencing) approaches have revealed low amounts of detectable divergent transcription at RNA polymerase II promoters [54,55]. Coincidentally, plant genomes harbor hundreds of transcription factors with Myb-like DNA-binding domains similar to Rap1 in yeast, while vertebrate genomes only contain a handful of Myb-like proteins. Myb is a conserved DNA binding protein found in retroviral oncogenes, and organisms ranging from sea urchins to humans [56,57]. Typically, these transcription factors control transcriptional responses to proliferation, differentiation, and environmental stresses [58]. It would be interesting to examine whether the expansion of Myb-related transcription factor gene families and the Myb domain specifically repress divergent transcription in plants and other organisms.

## Conclusion

In conclusion, stable binding of sequence-specific transcription factors to cis-regulatory elements can limit divergent noncoding transcription and thus control promoter directionality. Recent work highlights one way in which the information encoded in cis-regulatory elements can be interpreted by trans-acting regulatory proteins like transcription factors to produce a transcriptional output. It is possible that other sequence-specific transcription factors and DNA binding proteins limit cryptic transcription near regulatory elements, as shown for Rap1.
